# Development and psychometric testing of the Aesthetics of Everyday Life Scale in Aging (AELSA)

**DOI:** 10.1186/s12877-024-04874-w

**Published:** 2024-03-18

**Authors:** Fatemeh Sadat Izadi-Avanji, Nafise Zamani, Ismail Azizi-Fini, Esmaeil Mohammadnejad

**Affiliations:** 1 Department of Medical-Surgical Nursing, Trauma Nursing Research Center, School of Nursing and Midwifery, Kashan University of Medical Sciences, Kashan, Iran; 2 Department of Medical-Surgical Nursing, School of Nursing and Midwifery, Kashan University of Medical Sciences, Kashan, IR Iran; 3 Department of Intensive Care and Emergency Nursing, School of Nursing and Midwifery, Kashan University of Medical Sciences, Kashan, Iran; 4 Department of Medical-Surgical Nursing and Basic Sciences, School of Nursing and Midwifery, Tehran University of Medical Sciences, Tehran, Iran

**Keywords:** Aesthetics, Aging, Development, Life, Psychometrics, Questionnaire

## Abstract

**Background:**

The aesthetics of everyday life improves physical and mental health and social communication. This study aims to develop and test a novel instrument that assesses the aesthetics of the everyday life of older adults.

**Methods:**

A mixed-methods study with a sequential exploratory approach was conducted from November 2021 to December 2022. Item generation and questionnaire formation were developed through interviews with older adults and a literature review (stage 1). A cross-sectional study was then conducted to test the psychometric properties of the novel scale among 380 older adults referred to Urban Comprehensive Health Service Centers (stage 2). The construct validity was tested via exploratory factor analysis (EFA) and with the principal component analysis method. Internal consistency and reliability of the scale were evaluated with Cronbach's alpha and test–retest with a 2-week interval.

**Results:**

The initial scale was prepared with 39 items. In stage 2, EFA revealed a seven-factor model with 34 items. Internal consistency was acceptable for extracted sub-scales (Cronbach's alpha range: 0.67- 0.93) and the total score (0.926). The intra-class correlation coefficient for test–retest reliability was 0.90.

**Conclusions:**

The AELSA is a valid and reliable instrument for evaluating the aesthetics of everyday life in older adults. the scale will help policymakers in formulating interventions to improve mental health and well-being in older adults. Moreover, Further studies is need to provide more support of construct validity.

## Background

Aesthetics is a vast domain, and the aesthetics of everyday life is a part of that domain that rejects art-centered aesthetics. This domain points to the continuities between aesthetic experience and everyday experience [1, 2]. The aesthetics of everyday life fall outside the realm of aesthetic theory and study the aesthetic experience of objects and daily acts that are not traditionally referred to as aesthetic [3].

According to Kant's theory, there is no objective feature that beautifies an object or act, because the beauty of an object depends on one's judgment and imagination. Based on this theory, older adults have personal tastes or preferences that make up their daily lives [4]. Also, Nietzsche believes that attention to older adults' aesthetics of everyday life is required to enhance lives and justify reasons for them to continue living [5]. Hegel supports the idea that the aesthetics of older adults help them to enhance their social connection and find activities that interest them [6].

Older adults' desire to have successful aging and the aesthetics of everyday life motivates them to have aesthetic experiences in life [7]. There are several studies on the possible relationships between aesthetics and happiness or satisfaction with life [8–10], which signal that old people recognize the beauty standards of society and seek to achieve them [9]. Older adults gradually become inactive and require assistance from other individuals [6]. Therefore, they require a method that they can use to overcome such effects. The aesthetics of everyday life may be the answer to physical strains and disorders among the older population [11]. The aesthetics of everyday life enable older adults to maintain the appearance of the body, which results in high self-esteem, improved relationships with others, and physical and psychosocial health simultaneously [12, 13]. However, the aesthetics of everyday life debate among older adults has not yet been fully developed [11].

Healthcare providers, in addition to focusing on diseases and disorders, are committed to providing comprehensive care by understanding concepts related to human empowerment, well-being, and happiness [14]. They should develop tools for these concepts to assess the condition of older adults and perform the necessary interventions [15]. The literature review of the databases shows that, to date, no specific tools have been developed to measure the aesthetics of daily life for older people. Instruments such as Values in Action [16], the HEXACO Personality Inventory [17], and the Oxford Happiness Inventory [18] have limited items to measure aesthetic appreciation, which are not specific to older people.

Given the absence of a specific tool to measure the aesthetics of everyday life and the dependence of this concept on cultural and social contexts, designing a tool that captures older adults' aesthetics of everyday life is imperative. Thus, this study aims to develop the aesthetics of everyday life scale for the older adults and test its psychometric properties.

## Methods

### Design

A mixed-methods study with a sequential exploratory approach was conducted from November 2021 to December 2022. The study aimed to develop and test a novel instrument that assesses the aesthetics of the everyday life of older adults.

The aesthetics of everyday life scale for older adults was developed in two stages. First, items were generated with qualitative research and a literature review. Second, the scale's psychometric properties were tested among older adults referring to the Urban Comprehensive Health Service Centers (UCHSCs). A summary of the study steps and its results is presented in Table 3.

### Stage 1: Questionnaire construction

The items were extracted based on 16 semi-structured and face-to-face interviews on the topic of the aesthetics of everyday life for older adults. The items determined understanding of the aesthetic of life in several domains. Domains were "art as a source of peace"," environment beauty as the source of vitality, "spiritual beauty and the transcendence of the soul", "the family, and others synonymous with beauty, "fun and communication", "independence and living with dignity", and "beauty of appearance and physical health" in old age. In addition to interviews, potential items were also identified through a literature review and following up on three questionnaires, including the HEXACO Personality Inventory [17], the Values in Action [16], and the Oxford Happiness Inventory [18]. The item-generation process resulted in an initial scale, which included 61 items, and all items were formulated as a 5-point Likert–type scale that ranged from 5 = strongly agree to 1 = strongly disagree.

To maximize the qualitative face validity of the scale, a meeting was organized with the 10 older adults. They were asked to examine the scale items in terms of their difficulty, ambiguity in the meaning of words, and the relationship of the items with the questionnaire's purpose to improve scale items.

In addition, to determine quantitative face validity, the same older adults were asked to rate the importance of each item on a 5-point Likert scale. The impact score of items was calculated using the following formula: impact score = frequency × importance [19].

The qualitative and quantitative content validity was examined in a subsample (12 people). The scale was sent to experts (5 experts on the questionnaire design and methodology, four psychologists, and three gerontologists, and they were asked to comment on the grammar, wording, item allocation, and scoring of the scale items. Following the experts’ comments, changes were made to the wording of some items.

The quantitative validity of the scale was evaluated using the content validity ratio (CVR) and content validity index (CVI) [20]. Therefore, the same experts were asked to rate the necessity of each item on a 3-point scale (is not essential, is useful but not essential, and is essential 1 to 3, respectively). Then CVR was calculated based on the following formula: CVR = (Ne—N/2)/ (N/2), in which Ne is the number of experts who selected the "essential" option and N is the total number of experts. The numeric value of CVR was determined by Lawshe Table [21]. The items with CVR values greater than 0.56 were accepted.

To evaluate CVI, the same experts were asked to rate the relevancy of items on a 4-point Likert scale (very relevant = 4, relevant = 3, somewhat relevant = 2, not relevant = 1). The CVI was calculated by dividing the number of experts who selected “very relevant” or, “relevant” by the total number of experts. Items with values lower than 0.70 were deleted.

### Stage 2: Psychometric testing

A cross-sectional study was conducted to evaluate the construct validity of the 39-item scale among older adults referring to UCHSCs from November 2021 to December 2022. The inclusion criteria were older adults 60 years and older, residing in their own private homes, and not having cognitive impairment according to the Mini-Mental State Examination. The sample size for construct validity was calculated based on the number of scale items. Recommendations range from 2 to 20 participants per item [22]. In this study, 10 participants were considered per item on the scale.

In this study, the multi-stage cluster sampling technique was used to select older adults. There are 30 UCHSCs in Kashan. First, 35 percent of the centers (10 centers) were randomly selected. The researcher referred to selected UCHSCs, Kashan, and received a list of older adults 60 years and older based on their electronic record. Then, 390 older people were selected using the probability proportionate to the size sampling method.

### Instruments

In addition to the newly developed AELSA, the biographical information questionnaire was used to collect data. The participants were invited to UCHSCs by phone. First, they signed an informed consent form. Then, the questionnaires were given to the participants to fill out. For illiterate participants, the researcher read the items and wrote the answers in the questionnaire. For each of the older adults who refused to fill out the questionnaires, another sample was randomly selected from the same center.

### Scale’ Reliability and stability

Internal consistency of the AELSA was evaluated by Cronbach's alpha in a subsample (15 older adults). The test–retest reliability was performed on the same older adults at intervals of two weeks. The intraclass correlation coefficient (ICC) was estimated based on the absolute agreement specified and a 2-way mixed-effects model between baseline and follow- up [23].

The standard error of measurement (SEM) indicates the variation in the measurement errors for a test. The difference between the test score—retest score and its standard deviation (SD difference) was determined. Then SEM was calculated with the following formula: SEM = SD difference /√2) [19]. Furthermore, the Smallest Detectable Change (SDC) of AELSA was calculated with the following formula: (S*DC*95% = *SEM* × √2 × 1.96). The SDC is the minimum change that participants must show on the instrument with 95% confidence that the observed change is actual and not just a measurement error [23].

### Data analysis

Statistical analysis was performed using SPSS 16.0 (SPSS Inc., Chicago, IL, USA). The construct's validity was tested by applying exploratory factor analysis (EFA). Principal Component Analysis and the Varimax rotation method technique were applied to the extraction of factors. Principal components analysis (PCA) is technique to variable-reduction. PCA tests whether all the items included in the scale measure the construct. Namely it shows which items are not representative of the measured construct and should be removed from the scale. Principal components are a few linear combinations of the original variables that maximally explain the variance of all the variables [24]. Varimax rotation is an important step in Factor Analysis and Principal Component Analysis. Factor rotation results in a small number of important variables highlighted, which makes it easier to interpret results [25]. The Kaiser–Meyer–Olkin (KMO) test was used to determine the sampling adequacy of the data for exploratory factor analysis. The correlation in the data was determined by applying Bartlett's Test of Sphericity. Orthogonal factors with an Eigenvalue > 1.0 were required to explain a total variance > 60. Factor loadings less than 0.4 were suppressed. Cronbach's coefficient was used to evaluate the internal consistency of the scale. The ICC was used for test–retest reliability at a two-week interval. The results were considered significant at *p* < 0.05.

## Results

In the final analysis, 380 questionnaires were used to construct validity of the aesthetics of everyday life scale for older adults. Ten questionnaires were omitted because they were incomplete. Most of the participants (54.2%) were male. The participants were 60 to 92 years old. 76.3% of the participants were married, and 63.7% had an education in high school or lower. In terms of occupational, 41.8% of the participants were retired, and 40.5 were housekeepers. Furthermore, most of the participants (68.2%) had sufficient income. The demographic characteristics of older adults are shown in Table 1.

**Table 1:** Demographic characteristics of the older adults

Demographic information		Participants *n* = 380 (%)
Gender	Female	174(45.8)
	Male	206(54.2)
Marital status	Married	290(76.3)
	Single (single, divorced, widowed)	90(23.7)
Education	Illiterate	47(12.4)
	High school or lower	242(63.7)
	Diploma and above	91(23.9)
Occupation	Self-employee	37(9.7)
	Retired	159(41.8)
	Housekeeper	154(40.5)
	Disabled	30(7.9)
Income	Insufficient	121(31.8)
	Sufficient	259(68.2)

### Stage 1: scale construction

The item-generation process resulted in a preliminary scale of 61- items. The items were formulated from strongly agree = 5 to strongly disagree = 1.

### Face and content validity

As displayed in Table 3, in this stage, the items were evaluated in terms of difficulty, relevance, and ambiguity by ten older people. The results indicated the relevance of items with the questionnaire's purpose and not having ambiguity in understanding the items. Two items were merged due to semantic overlap. Eight items had an impact score of ≤ 1.5 and were deleted [19].

### Content validity

The qualitative content validity was examined based on the 12 experts' opinions, and suggestion modifications were applied to the nine items. According to Lawshe's table, CVR was accepted for 45 items with a coefficient value > 0.67. The average CVI (S-CVI/Ave) was = 0.94, indicating excellent content validity of the AELSA. More information is shown in Table 3 [26].

## Construct validity

Construct validity was evaluated using data from a large sample of older adults (*n* = 380). EFA for the AELSA was performed using Principal Component Analysis (PCA) with a Promax rotation method. Seven factors with an eigenvalue of > 1.0 were extracted based on the Kaiser–Guttman rule. The KMO (0.0.84) and Bartlett's test of Sphericity (7028.663; df = 561; *p* < 0.001) indicated acceptable sampling adequacy. Factor loadings ≥ 0.40 were considered for each factor. The run of EFA led to the removal of 3 cross-loading and low-loading (< 0.400) items. Based on the conducted PCA, the AELSA included a total of 34 items over 7 components. The results of the factor analysis are shown in Table 2**.**

**Table 2:** AELSA factor analysis and internal reliability

	**Item including in the factor**	**Factors Loadings**						
No		**1**	**2**	**3**	**4**	**5**	**6**	**7**
**1: Family and others**								
Q18	Communication with my children and grandchildren makes my life beautiful	0.781						
Q17	Solving the problems of my family members makes me feel beautiful	0.756						
Q23	The health of my family members and others is one of the most important beauties of my life	0.756						
Q19	Having a competent and successful child is one of the beauties of my life	0.755						
Q22	The intimate relationships between my family members build beautiful moments in my life	0.731						
Q20	The happiness of my family and others makes me feel calm	0.716						
Q25	Respect for my dignity by family members gives me a sense of vitality	0.545						
Q21	Living with my wife is one of the beauties of my life	0.481						
**2: Art and artistic activities**								
Q5	I experience a good feeling by looking at works of art (paintings, calligraphy, etc.)		0.882					
Q3	I like to do some artwork (sewing, painting, working with wood, etc.)		0.858					
Q4	By Creating artwork (painting, photography, carpet weaving, tailoring, etc.) I created beautiful moments for myself		0.856					
Q1	Listening to music is enjoyable for me		0.775					
Q2	Reading or hearing the poems of Great Poets is lovely to me		0.645					
**3: Communication and social presence**								
Q27	Participating in group activities (sports) creates beautiful moments for me			0.812				
Q26	Attending group entertainment gives me a sense of beauty			0.762				
Q29	Communicating with young people creates beautiful moments for me			0.695				
Q28	Communicating with my peers creates beautiful moments for me			0.621				
Q30	Commuting with my family and others makes my life beautiful			0.554				
**4: Spirituality and transcendence of the soul**								
Q10	Pilgrimage trips create a sense of beauty in me				0.910			
Q12	Helping others creates a beautiful spiritual feeling				0.900			
Q11	Doing religious activities (praying, going to religious places, participating in religious ceremonies,) makes my moments beautiful				0.718			
Q9	Observance of human virtues (respecting others, contentment, forgiveness, honesty, etc.) makes my life beautiful				0.688			
Q29	The moment of my relationship with God gives me peace				0.484			
**5: Beauty of appearance and physical health**								
Q13,	Maintaining my fitness gives me vitality and peace					0.885		
Q36	Maintaining and promoting my physical health makes my life beautiful					0.826		
Q37	Maintaining the beauty of my face makes me feel good					0.788		
Q38	Having a clean and tidy appearance is one of the beauties of my daily life					0.677		
**6: Independence in life**								
Q35	Being able to do my daily chores makes my life beautiful						0.764	
Q33	Financial independence makes my life beautiful						0.681	
Q32	Independence in decision-making adds to the beauty of my life						0.626	
Q31	Being able to solve my problems is one of the beauties of my life						0.611	
**7: Perception of the environment's beauty**								
Q24	My living environment's beauty invigorates me							0.902
Q6	Gardening and growing flowers and plants make beautiful moments of my life							0.848
Q7	My surfing in nature creates beautiful moments for me							0.630
		1	2	3	4	5	6	7
Eigenvalue		8.48	3.40	2.92	2.17	2.12	1.71	1.32
Percentage of variance explained		24.931	9.991	8.592	6.374	6.218	5.016	3.866
Total percentage of the factor model		64.98						
Cronbach’s alpha per factor		0.93	0.80	0.67	0.86	0.78	0.79	0.88

### Internal consistency and test re-test reliability

As displayed in Table 2, Internal consistency using Cronbach's alpha, for the 34 items of the scale was high (0.926), indicating that some questions may be redundant. Alpha values between 0.70 and 0.90 are considered excellent, whereas values < 0.70 indicate inconsistency, and values > 0.90 indicate redundancy of items [27].

The AELSA had excellent reliability (ICC = 0.90, 95% CI = 0.85–0.95). The ICC estimates above 0.8 as excellent, between 0.6 and 0.79 as strong, between 0.4 and 0.59 as moderate, and below 0.4 as poor [28]. Furthermore, the calculated values of SEM and SDC were 1.38 and 3.81, respectively (Table 3).

**Table 3:** Development of candidate items

**Stage 1. Questionnaire construction**		
**Qualitative study (*n* = 16) Literature review**	**Item generation**	**61 items developed**
**Evaluation by older adults (*n* = 10)**	**Face validity**	**1) Qualitative face validity:** Two items were merged due to semantic overlap, 6 items amended
		**2) quantitative face validity:** Impact score of 8 items < 1.5 and were deleted
**Evaluation by expert (*n* = 12)**	**Content validity**	**1) Qualitative Content validity:** 5 items amended **2) Content Validity Ratio (essential):** 6 items had CVR < 0.56 and were deleted^a^ **3) Content Validity Index** (relevancy): I-CVI value^b^ of 3 items < 0.70. and were deleted S-CVI/Ave = 0.94^c^
**Stage 2. Evaluating psychometric properties of the scale**		
**Main study (*n* = 380)**	**Structural validity**	**7-Fctor model** -Family and others (8 -items) -Art and artistic activities (5–items) -Communication and social presence (5 items) -Spirituality and transcendence of the soul (5 –items) -Beauty of appearance and physical health(4–items) -Independence in life (4 -items) -Perception of the environment's beauty (3–items)
		**EPEA-S** = 34 items (final version)
**Scale’ Reliability and stability**		
**The sub-sample (*n* = 15)**	**Internal consistency**	Cronbach’s alpha = 0.926
**The sub-sample (*n* = 15)**	**Test–retest reliability**	ICC = 0.90, 95% CI = 0.85–0.95^d^ SEM agreement = 1.38^e^ SDC_95_ = 3.81^f^

^a^*CVR *Content Validity Ratio

^b^*I-CVI *Item Content Validity Index

^*c*^*S-CVI/Ave *Scale Content Validity Index/Average

^d^*ICC *Intra-class Correlation Coefficient

^e^*SEM agreement *Standard Error of Measurement

^f^*SDC*_*95*_ Smallest Detectable Change

## Discussion

The study developed a novel scale to measure the aesthetics of everyday life based on the experiences of older adults in an Iranian population aged 60 and older. Development and validation of the AELSA were performed using qualitative and quantitative methods and demonstrated that the scale is valid, consistent, and reliable. The EFA showed an explained variance of acceptable for scale that confirms its ability to measure the concept of aesthetics of everyday life in older adults.

The EFA results showed the highest percentage of variance explained was related to the subscale of family and others. The literature review supported this finding that family support, family interactions and relationships, and family structures, play an important role in maintaining the physical, mental, and social health of older adults and increases their readiness to face life stresses in Asian communities [29]. A study in China revealed that communicating with relatives by phone improves older rural adult’s mental health [30]. Expressing devotion and affection to older adults can help them feel valued [31, 32]. Interaction with others result in stimulating brain cells, improving cognitive function, reducing the risk of dementia, and improving quality of life among older adults [33, 34]. It seems social interactions and family relationships, as an aesthetic experience affect an individual mental and social health by instilling a sense of self-efficacy.

The percentage of variance explained for five subscales (Art and artistic activities, Communication and social presence, Spirituality and transcendence of the soul, Beauty of appearance and physical health, and Independence in life) were almost close together. These results indicate that the importance of other aspects of esthetics are almost equal in older adult life. A qualitative study revealed that aesthetic experiences of older adults is incorporated in three domains include in maintaining independence, significant others’ connectedness, and experiences that lead to inner peace [2]. Highmore (2011) argues that aesthetics of the everyday have an extraordinary power that affects all aspects of older adults’ everyday life and guides their actions in the best possible way. However, it is often unrecognized [35]. For example, art activities (participatory and receptive) as an aesthetic experience can improve memory, lower stress levels, help maintain social engagement, and offer a therapeutic tool for physical and psychological well-being in older adults and vulnerable individuals [36–38]. Jadidi et al. (2021) argue that older adults use religious coping strategies to express their spiritual needs (meaning, hope and peace) and to get out of distress which leads to a degree of their spiritual awakening. Hence, spirituality and transcendence of the soul is considered as aesthetics of everyday life among older adults [39].

In the present study, beauty of appearance and physical health subscales also accounted for a high percentage of variance. The body is a significant aspect of a person’s identity that helps older adults to interact with various environment [40, 41]. Body aesthetics enables older adults to understand the inner and outer aspects of their bodies [40–42], and develop their perceptions, behaviors and thoughts. When everyday life is considered as a form of aesthetics, the body can also be regarded as an aesthetic experience for older adult. Therefore, older adults try to have a living, sensitive, dynamic and perceptual body [40–43],

The present study has several strengths. Development of the everyday aesthetics concept and the item-generation process were done by integrating both deductive and inductive approaches. This could help to develop a comprehensive scale. Furthermore, face validity was evaluated by the target group to ensure that AELSA is understandable, and measures the intended structure and concept. The study samples were drawn with a multi-stage random sampling method from the general older adult population. Nevertheless, further psychometric testing in different regions and cultures on the aging population is warranted to prove the reliability and stability of the AELSA. The study faced limitations that open avenues for further research. It was not possible to measure discriminant validity as subcategories of construct validity due to the limited study time. Therefore, it is suggested that another study be conducted to assessing construct validity with confirmatory factor analysis and discriminant validity to provide more support for construct validation [44]. In addition, the participants in the study were older adults. The number of items on the developed scale (AELSA) and the background information questionnaire were large. In order to increase the accuracy of the older adults in answering the questions and to prevent their stress, it is suggested evaluating the concurrent validity in further studies.

## Conclusion

The AELSA is the first scale designed to measure the aesthetics of everyday life in older adults. The scale has acceptable stability, reliability, and validity and could be used as the assessment tool for understanding the level of older adults' aesthetics of everyday life. This scale can help older adults enhance their inner peace, and maintain their well-being and independence in front of everyday challenges. In addition, the scale will help policymakers in formulating interventions to improve mental health and quality of life in older adults.

## Data Availability

No datasets were generated or analysed during the current study.

## Abbreviations

UCHSC: Urban Comprehensive Health Service Centers
AELSA: Aesthetics of Everyday Life Scale in Aging
EFA: Exploratory Factor Analysis
CVR: Content Validity Ratio
CVI: Content Validity Index
ICC: Intraclass Correlation Coefficient
SEM: The standard error of measurement
SD: Standard deviation
SDC: Smallest Detectable Change
KMO: Kaiser–Meyer–Olkin
PCA: Principal Component Analysis
